# pH and Antimicrobial Activity of Portland Cement Associated with Different Radiopacifying Agents

**DOI:** 10.5402/2012/469019

**Published:** 2012-10-16

**Authors:** Juliane Maria Guerreiro-Tanomaru, Ana Lívia G. Cornélio, Carolina Andolfatto, Loise P. Salles, Mário Tanomaru-Filho

**Affiliations:** Department of Restorative Dentistry, Araraquara Dental School, São Paulo State University (UNESP), 14801-385 Araraquara, SP, Brazil

## Abstract

*Objective*. The aim of this study was to evaluate the antimicrobial activity and pH changes induced by Portland cement (PC) alone and in association with radiopacifiers. *Methods*. The materials tested were pure PC, PC + bismuth oxide, PC + zirconium oxide, PC + calcium tungstate, and zinc oxide and eugenol cement (ZOE). Antimicrobial activity was evaluated by agar diffusion test using the following strains: *Micrococcus luteus*, *Streptococcus mutans*, *Enterococcus faecalis*, *Pseudomonas aeruginosa*, and *Candida albicans*. After 24 hours of incubation at 37°C, inhibition of bacterial growth was observed and measured. For pH analysis, material samples (*n* = 10) were placed in polyethylene tubes and immersed in 10 mL of distilled water. After 12, 24, 48, and 72 hours, the pH of the solutions was determined using a pH meter. *Results*. All microbial species were inhibited by the cements evaluated. All materials composed of PC with radiopacifying agents promoted pH increase similar to pure Portland cement. ZOE had the lowest pH values throughout all experimental periods. *Conclusions.* All Portland cement-based materials with the addition of different radiopacifiers (bismuth oxide, calcium tungstate, and zirconium oxide) presented antimicrobial activity and pH similar to pure Portland cement.

## 1. Introduction

The ideal root-end filling material should present certain characteristics, such as ability to seal the root canal system, dimensional stability in the presence of humidity, and radiopacity. Equally important are its ability to induce repair, antimicrobial action, and biocompatibility. All these properties contribute towards the success of endodontic surgery [1].

Since its introduction as a root-end filling material in 1993, the clinical applications of mineral trioxide aggregate (MTA) have been expanded. Presently, MTA is also used as a reparative cement due to its alkaline pH [2]. The mechanism of action of MTA is similar to that of calcium hydroxide. However, the manipulation and insertion of this cement into retrograde preparations are extremely difficult. Yet another disadvantage of MTA is its high cost [3].

Several studies have evaluated Portland cement (PC) as an alternative to MTA [4, 5]. One of the limitations of PC is its low radiopacity, requiring addition of a radiopacifier prior to use. Bismuth oxide, the radiopacifying agent present in MTA, is not considered ideal by some authors. A number of studies have shown that this radiopacifier interferes with the mechanical stability of the cement by increasing its porosity [6] and also that it may negatively affect MTA's biological properties [7]. With this in mind, alternative radiopacifiers have been investigated. Bortoluzzi et al. [8] have studied several radiopacifying agents (bismuth oxide, barium sulfate, iodoform, and zirconium oxide) that may be combined with PC. Gomes Cornélio et al. [9] investigated the association of different radiopaque substances with PC using Murine periodontal ligament cells (mPDL) and rat osteosarcoma cells (ROS 17/2.8) and did not observe evidence of cytotoxicity when the cement was mixed with bismuth oxide, zirconium oxide, or calcium tungstate.

Therefore, promising alternatives as radiopacifying agents for PC have been proposed. Now, it is essential to assess how these associations might affect other important properties of sealing cements, such as pH and antimicrobial action. Antimicrobial action and ability to induce formation of mineralized tissue are both dependent on alkaline pH. These properties have already been thoroughly investigated in pure PC [10].

The aim of the present study was to evaluate and compare the antimicrobial action and pH changes promoted by PC alone and in association with different radiopacifying agents (bismuth oxide, calcium tungstate, and zirconium oxide).

## 2. Materials and Methods

### 2.1. Agar Diffusion Assays

The materials and associations investigated are presented in Table 1. PC and associations were manipulated on sterile glass slabs at a ratio of 1 g of powder to 320 *μ*L of water. ZOE cement was manipulated at a ratio of 1 g of powder to 0.2 g of eugenol. 

Antimicrobial activity was evaluated by agar diffusion test using different microorganisms (Table 2). Inocula of each strain were prepared by suspending cells after growth on plates with Brain Heart Infusion Agar or Sabouraud broth (*C. albicans*). Then, they were incubated at 37°C for 24 hours.

Evaluation of the antimicrobial activity was carried out by the agar well diffusion method, using the double agar layer technique. All assays were conducted in triplicate. A base layer was prepared by placing 12 mL sterile culture medium cooled to 50°C onto sterile Petri dishes measuring 15 × 150 mm. The seed layer was prepared by suspending the inoculum in culture medium at 50°C to a final concentration of 10^6^ CFU/mL. Once the base layer had solidified, 8 mL of the seed layer were added to the dishes. 

After solidification of the seed layer, wells were formed by punching holes on the agar using sterile aluminum cylinders measuring 4 mm in diameter. The placement of the holes was equidistant and 15 mm from the plate edge. Wells were then completely filled with PC and the associations of PC with the different radiopacifiers to be evaluated.

The plates were kept at room temperature for 2 hours to allow prediffusion of the materials, then incubated at 37°C for 24 hours. Following the incubation period, aliquots (5 mL) of agar TTC (triphenyl tetrazolium chloride—Merck KgaA, Darmstadt, Germany) gel were added to aid the identification of viable cells and allow optimal visualization of the zones of inhibition. Agar TTC was prepared by mixing liquified agar (Difco) with 0.05% TTC to a concentration of 1.0%. After being added to the plates, the gel was allowed to solidify and plates were incubated once again for 30 minutes at 37°C.

Images of the well-illuminated Petri dishes against a blue background, to contrast with the red color of the viable colonies, were digitized, and the diameters of the zones of inhibition around each well were measured using the Image Tool software (UTHSCSA Image Tool for Windows, version 3.0).

### 2.2. Evaluation of the pH

For the pH analyses, 10 standardized polyethylene tubes measuring 10 mm in length and 1.5 mm in diameter were prepared for each material tested, totaling 50 tubes. The materials tested were pure Portland cement (PC, Votorantim, SP, Brazil), PC in association with different radiopacifiers: PC + bismuth oxide, PC + zirconium oxide, and PC + calcium tungstate (all radiopacifying agents were obtained from Sigma Aldrich, St Louis, MO), and zinc oxide and eugenol cement (ZOE).

Immediately after manipulation of the materials, the tubes were filled and both ends were wiped. Then, each tube was radiographed to ensure adequate and complete filling with the materials. After that, tubes were placed in lidded flasks (JProlab, São José dos Pinhais, PR, Brazil) containing 10 mL of distilled water with neutral pH (pH previously measured = 6.5) and maintained at 37°C. After 12, 24, 48, and 72 hours, the water pH was measured using a DMPH-2 pH meter (Digimed, São Paulo, SP, Brazil). The device was previously calibrated using buffer solutions with pH 4, 7, and 10. Throughout the experiment, the pH meter was periodically recalibrated using the same solutions. Measurements were conducted in an environment with controlled and constant temperature of 25°C. The pH of the water from a flask containing only distilled water and an empty tube was measured at all experimental periods.

Results were subjected to a normality test and, subsequently, to ANOVA for comparisons among the different groups and to Tukey's multiple comparisons test. The significance level was set at 5%.

## 3. Results

### 3.1. Agar Diffusion Assays

The results demonstrated that all microbial species were inhibited by the cements evaluated. Generally, the inhibition haloes were lower for *E. faecalis*, *S. mutans*, and *P. aeruginosa* (except for ZOE). The means and standard deviations observed for the antimicrobial action of each material are presented in Table 3.

### 3.2. Evaluation of the pH

The means and standard deviations for the pH values of the cements at each experimental period are presented in Table 4. The results demonstrated that all radiopacifying agents tested promoted pH increase similar to pure Portland cement (mean 10.2) and that addition of radiopacifiers to PC did not affect this property. ZOE presented the lowest pH values at all experimental periods. 

## 4. Discussion

The agar diffusion test (ADT) is commonly used to assess the antimicrobial action of dental materials [11]. This method allows evaluation of the antimicrobial properties of different substances (cements, intracanal medications, and irrigating solutions, among others) against a large number of microbial strains, at various concentrations. The microorganisms used in the present work have been implicated in cases of persistent infection or treatment [12]. Nagayoshi et al. [13] observed the presence of *E. faecalis* was 40.6% in root canals from 32 adults undergoing retreatment for periapical lesions after endodontic treatment performed at least 2 years previously.

Ozbek et al. [14] investigate the presence of *E. faecalis* in primary endodontic infections and failed endodontic treatment, suggesting the presence of this microorganism in not less than 61% of all endodontic infections.

Important factors should be taken into consideration when evaluating materials using the agar diffusion test, such as the physicochemical properties of the material; its speed and rate of diffusion [15]; the concentration of the antimicrobial agent; the composition, pH, and thickness of the culture medium. Moreover, this method requires careful standardization of inoculum density, culture medium contents, agar viscosity, and number/size of the specimens present on each dish [16]. Other disadvantages of this test are the need for substances to be diffused in agar prior to analysis and the difficulties of measuring the zones of microbial growth inhibition [17]. Furthermore, this technique presents relatively low sensitivity and is semiquantitative, being unable to distinguish between bactericidal and bacteriostatic properties of the materials [18]. Despite these limitations, ADT is a useful method for preliminary evaluation of the antimicrobial effect of different substances.

Preincubation for 2 hours allows substances to diffuse in the agar gel, producing zones of microbial growth inhibition [19]. Triphenyl tetrazolium chloride (TTC), which was added to the culture medium,is a redox indicator of cellular respiration in growing microorganisms. With the addition of TTC, the medium turns red in the presence of viable microorganisms that grew on the plates [20], facilitating the visualization of zones of inhibition.

Our results show that all materials evaluated display antimicrobial action, producing zones of inhibition against all microbial strains. The samples of Portland cement combined with radiopacifying agents did not significantly differ from each other or from pure PC, confirming that none of the radiopacifiers interfered with this property. In the present study, zinc oxide and eugenol cement was used as positive control of antimicrobial activity against all strains. The antibacterial action of this cement is related to the presence of eugenol [21], which presents powerful bactericidal action by inhibiting cell growth and respiration, even at low concentrations. However, at high concentrations, eugenol is cytotoxic, inhibiting white cell chemotaxis, synthesis of prostaglandins, and nerve activity [22].

Portland cement-based materials are basically calcium oxides, which form calcium hydroxide when mixed with water. Calcium hydroxide is known to induce pH rise by dissociation of calcium and hydroxyl ions, as demonstrated by Hungaro Duarte et al. [23]. Other investigations have linked the antimicrobial action of MTA to its high pH [24]. However, it has been demonstrated that several endodontic microorganisms may be killed under conditions that are not pH mediated [25].

All samples of pure Portland cement, as well as PC combined with radiopacifying substances, promoted alkaline pH in all experimental periods (12, 24, 48, and 72 hours), with no statistical differences between the groups. This finding indicates that none of the radiopacifiers affected this property of PC. Camilleri [26] reported similar results for Portland cement combined with different radiopacifying substances, such as barium sulfate (BaSO_4_), gold (Au), and silver/tin (Ag/Sn), and observed that none of these agents affected the alkalinizing properties of pure Portland cement.

pH values near 12 are known to inhibit the activity of several microorganisms, including resistant bacteria such as *Enterococcus faecalis* [27]. Despite the fact that smaller zones of inhibition were observed for this strain, antimicrobial activity against *E. faecalis* was still detected for all cements tested, similarly to the results reported by Estrela et al. [28]. Contrastingly, other authors have reported the lack of antibacterial activity of Portland cement and of MTA against *E. faecalis* [22, 29]. In an attempt to enhance the antimicrobial action of MTA, replacement of the water in the mixture with other liquids, such as 2% chlorhexidine, has been suggested [30]. Addition of antimicrobial substances to MTA is likely to improve its antibacterial action, but might negatively affect other properties of the cement [2]. A recent study [31] showed that association of MTA with silver-zeolite improves the antimicrobial action of MTA against several bacterial strains, including *E. faecalis.* However, as mentioned above, it is important to investigate whether these substances affect the physicochemical properties of the cement. Moreover, the limitations of *in vitro* testing of antimicrobial agents should be taken into consideration.

## 5. Conclusion

Considering the methodology employed and the results obtained, it was concluded that the addition of radiopacifiers (bismuth oxide, calcium tungstate, and zirconium oxide) to Portland cement did not interfere with its antimicrobial action and pH. All associations evaluated presented similar results among each other and in comparison with pure Portland cement. 

## Figures and Tables

**Table 1:** Composition and manufacturer of the materials used in this study.

Material	Composition	Manufacturer
Portland	Tricalcium silicate, dicalcium silicate, iron-calcium aluminate, calcium sulfate, tricalcium aluminate, calcium carbonate, magnesium oxide, calcium oxide	Votorantim, SP, Brazil
Bismuth oxide	Bismuth oxide	Sigma Aldrich, St Louis, MO
Zirconium oxide	Zirconium oxide	Sigma Aldrich, St Louis, MO
Calcium tungstate	Calcium tungstate	Sigma Aldrich, St Louis, MO
Zinc oxide and eugenol	Powder: ZnO	S.S.White Art. Dent. Ltda., Rio de Janeiro, RJ, Brazil
	Liquid: C_10_H_12_O_2_	

**Table 2:** Strains used as indicator of antimicrobial activity, their source, and morphotype.

Microorganisms	Source	Morphotype
*Micrococcus luteus *	ATCC 9341	Gram-positive cocci
*Streptococcus mutans *	ATCC 25175	Gram-positive cocci
*Enterococcus faecalis *	ATCC 29212	Gram-positive cocci
*Pseudomonas aeruginosa *	ATCC 27853	Gram-negative bacilli
*Candida albicans *	ATCC10231	yeast

**Table 3:** Means and standard deviations of the inhibition zones values (in millimeters)*.

	PC	PC + OBi	PC + OZr	PC + CT	ZOE
*E*. *faecalis *	9 ± 0	8 ± 0	8 ± 0	8 ± 0	10 ± 0
*P*. *aeruginosa *	12 ± 0	12.3 ± 0.57	10.6 ± 2.30	11 ± 1.73	24.6 ± 1.15
*C*. *albicans *	23.6 ± 1.15	24 ± 1	22.3 ± 0.57	22.3 ± 0.57	25.6 ± 2.08
*S*. *mutans *	11 ± 0	10.6 ± 0.57	10.6 ± 0.57	10.3 ± 0.57	12 ± 1
*M*. *luteus *	31.6 ± 0.57	31.3 ± 0.57	30 ± 1	30 ± 1	34.3 ± 1.15

*Means of the triplicate assays.

PC: Portland cement, OBi: bismuth oxide, OZr: zirconium oxide, CT: calcium tungstate, ZOE: zinc oxide and eugenol cement.

**Table 4:** Means and standard deviations of pH values for the different materials and periods.

	PC	PC + OBi	PC + OZr	PC + CT	ZOE
12 h	10.24 ± 0.05^A^	10.20 ± 0.21^A^	10.21 ± 0.12^A^	10.23 ± 0.07^A^	7.484 ± 0.41^B^
24 h	10.20 ± 0.08^A^	10.21 ± 0.17^A^	10.20 ± 0.13^A^	10.24 ± 0.06^A^	7.444 ± 0.34^B^
48 h	10.23 ± 0.08^A^	10.30 ± 0.15^A^	10.22 ± 0.14^A^	10.26 ± 0.08^A^	7.346 ± 0.31^B^
72 h	10.22 ± 0.08^A^	10.28 ± 0.17^A^	10.21 ± 0.13^A^	10.23 ± 0.10^A^	7.325 ± 0.30^B^

Same letters within the same period of time indicate statistically similar results (*P* > 0.05).

PC: Portland cement, OBi: bismuth oxide, OZr: zirconium oxide, CT: calcium tungstate, ZOE: zinc oxide and eugenol cement.
